# Ethical challenges in the treatment of non-refugee migrants with tuberculosis in Canada

**DOI:** 10.1093/pubmed/fdaa222

**Published:** 2020-12-15

**Authors:** Diego S Silva, Victoria J Cook, James C Johnston, Jennifer Gardy

**Affiliations:** Sydney Health Ethics, Faculty of Medicine and Health, University of Sydney, Camperdown, NSW 2050, Australia; Marie Bashir Institute for Infectious Diseases and Biosecurity, University of Sydney, Camperdown, NSW 2050, Australia; TB Services, Clinical Prevention Services, BCCDC, Vancouver, BC V5Z 4R4, Canada; Division of Respiratory Medicine, Department of Medicine, University of British Columbia, Vancouver, BC V6T 1Z4, Canada; TB Services, Clinical Prevention Services, BCCDC, Vancouver, BC V5Z 4R4, Canada; Division of Respiratory Medicine, Department of Medicine, University of British Columbia, Vancouver, BC V6T 1Z4, Canada; Surveillance, Data, and Epidemiology, Bill and Melinda Gates Foundation, Seattle, WA 98109, USA

**Keywords:** bioethics, high-income countries, immigration, justice

## Abstract

While attention to the ethical issues that migrants face in accessing tuberculosis care has increased in the last few years, most of the attention has focused on challenges that refugees face when emigrating. Less attention has been given to ethical challenges that arise in the context of providing tuberculosis treatment and care to non-refugee migrants in high-income countries (HIC), particularly those that do not face immediate danger or violence. In this paper, we analyze some of the ethical challenges associated with treating migrants with tuberculosis in the Canadian context. In particular, we will discuss (i) inter- and intra-jurisdictional issues that challenge quotidian public health governance structures, and (ii) the ethical imperative for the Canadian government and its provinces to clearly differentiate access to healthcare from a person’s immigration status to help overcome power imbalances that may exist between public health workers and their clients. The arguments presented herein could potentially apply to other HIC with some form of universal health coverage.

## Introduction

Recently, the plight of migrants living with tuberculosis (TB) has received attention and consideration by policy makers, academics, and the public alike.^1^^,^^2^^,^^3^ In parallel but unrelated fashion, the last decade has seen a renewed interest in analyzing what ethical obligations, if any, countries and societies have with regards to the health and healthcare of migrants within their own borders.^4^^,^^5^^,^^6^ Bringing together these two disparate fields, scholars have begun to turn their attention to the ethical challenges faced by migrants who have TB and the countries to which they have immigrated or are immigrating. Much of this consideration has focused on migrants who have fled dangerous situations to seek prosperity in high-income countries (HIC), e.g. Syrian refugees migrating to the European Union to escape war and genocide.^7^ Given the global rise in right-wing nationalism within HIC that is present in 2020, coupled with the difficulties of treating TB in many low-income migrant populations,^7^^,^^8^ especially refugee populations, it is reasonable and important to consider what justice requires of countries in which such migrants land. However, despite being less dire, the ethical challenges associated with the treatment of non-refugee migrants for TB is also important; not addressing such challenges endangers lives and may potentially affect the public’s trust in public health.

In this paper, we will focus our discussion on ethical challenges faced by persons and populations who immigrate to HIC for reasons beyond war or fear, e.g. non-refugees. In particular, we will focus on and analyze two categories of problems faced by migrants with TB in Canada: interprovincial jurisdictional challenges; and navigating power imbalances between healthcare workers representing the public health system and migrants with TB. First, we will give a brief overview of the ethics literature as it relates to health and migrants, particularly as it relates to those persons with TB. Next, we will describe the situation for migrants with TB in Canada. Finally, we will introduce two types of ethical challenges faced by healthcare workers in Canada working with migrants and TB and provide some preliminary guidance where possible. Before proceeding, we note that the ethics literature discussed in section one of the paper refers to the responsibilities of governments and societies in HIC, broadly speaking, which would then apply to different contexts; we then apply it in sections two and three in the context of Canada specifically, before speaking of the applicability of our analysis to other HIC in the conclusion.

## Ethics and migrants’ health

To begin, the International Organization for Migration (IOM) defines a ‘migrant’ as ‘… any person who is moving or has moved across an international border or within a State away from their habitual place of residence, regardless of (1) the person’s legal status; (2) whether the movement is voluntary or involuntary; (3) what the causes for the movement are; or (4) what the length of the stay is’.^9^ For the limited purposes of this paper, and as noted in the introduction, we will use the term ‘migrant’ in the context of Canada, and in particular people moving into Canada, and will exclude persons who are moving within Canada or these categorized as ‘refugees’ upon entry to Canada, i.e. someone who leaves their country due to fear of persecution because of ‘… race, religion, nationality, membership of a particular social group or political opinion …’.^10^ We note that the term ‘migrant’, as we are using it here, may not be its only possible use or that there are potentially better terms from which to choose; however, for the sake of simplicity and consistency we will use the IOM’s understanding of migrant.

Most ethics scholars argue that those societies and governments in HICs have some kind of obligation to care for the health and well-being of migrants, within one’s borders, while those in lower- and middle-income countries also have obligations that may be somewhat tempered by local restriction of resources.^5^^,^^6^^,^^11–13^ Various arguments have been forwarded to justify this claim from two of the primary categories of ethics theories. To begin, perhaps the most straightforward can be generated by theories that can be described as, broadly speaking, consequentialist; these theories claim that we ought to maximize the well-being of persons in the world, however one chooses to define ‘well-being’. Another traditional category of ethics theories are those of deontology, which generally hold that humans have an intrinsic dignity and sense of agency or autonomy that means we must always treat people as end-in-themselves, i.e. not as mere means to get what we want from others. As such, persons in extreme positions of need, e.g. such as those suffering from famine, require having their basic needs met so as to uphold and promote their dignity and autonomy.^14^ Given that this paper is not the place to expand upon these category of theories and the differences therein, it suffices to state under both broad accounts, unmet health needs should be addressed by states regardless of who has the health need and regardless of whether or not they are a citizen, either by appealing to upholding their dignity or by appealing to our obligation to maximize well-being. Stated differently, from an ethics viewpoint, we would argue that the need to provide care, and healthcare in particular, to migrants in HIC is conceptually overdetermined.

Other, more recent, political accounts as to the ethical obligation of HICs toward the good health of migrants are grounded in: healthcare being understood as a primary good necessary for human flourishing^7^; healthcare as a global public good, wherein states and societies have collective obligations to provide for such a good on the grounds of not exposing persons to preventable harms^13^; and healthcare for migrants being understood as a condition of fulfilling our obligations of global solidarity, wherein we acknowledge the interrelated and interdependent nature of persons globally that give rise to reciprocal obligations of care.^12^ All of these arguments can be grouped under the broad heading of cosmopolitanism, which holds that ‘… every person has global stature as the ultimate unit of moral concern and is therefore entitled to equal respect and consideration no matter what her citizenship status or other affiliations happen to be’.^5^

Although there is overwhelming agreement that the health needs of migrants should be addressed, reasonable and persistent disagreement exists with regards to the extent of such an obligation on the part of HICs, and how such an obligation ought to be discharged.^5^ Even if we believe that we have obligations toward promoting the good health of migrants, one might hold that we have obligations to care for the health and well-being of fellow citizens of our country, first and foremost. This is not to argue that the health of migrants does not matter, but that we ought to prioritize the health of our fellow citizens and permanent residents because of a shared history and current legal standing, agreeing to pay taxes for public or common goods, and participating in political and civic life of a region. For the purposes of this paper, we can acknowledge and set aside these important considerations; given the wealth of Canada and the low-cost of treating TB relative to other clinical treatments, while acknowledging that public funds are certainly limited, questions stemming from resource constraints do no often enter serious debate in the context of addressing infectious diseases, as is the case with treating active TB (with perhaps some exceptions—e.g. latent tuberculosis infection [LTBI] screening). While there may be some proportion of Canadians who might question the worthiness of spending money on migrant health, there is no strong evidence to suggest that Canadians actually believe we have no obligations whatsoever toward ensuring migrant health. For example, a recent survey of Canadians suggests quite positive overall perception of immigration, migrants and refugees, a viewpoint which has increased in 2020 when compared with 2019 trends.^15^

The existing ethics literature with regards to migrants and TB, given that it is nascent, has focused on arguing and advocating for access to healthcare on the part of migrants. Starting from the value of solidarity, the World Health Organization’s ‘Ethics Guidance for the Implementation of the End TB Strategy’ states that all migrants, regardless of status, ought to receive prompt and best-available TB care. It follows that TB care ought not to be withheld in cases where migrants are unable to pay and that work permits ought not to be denied to migrants who have tested positive for latent TB infection.^16^ Wild and colleagues, in turn, argue that the global health ethics literature supports the position that HICs are not only responsible for the well-being of migrants within their own borders, but that HICs bear some responsibility for improving the health systems and infrastructures in low-and-middle income countries, which are those that often see emigration in higher volumes than HICs and often to HICs themselves.^7^

Critically, Wild and colleagues also describe and support the ‘firewall argument’, namely that governments in countries that receive a high number of migrants, like HICs, ought to assure their migrant communities that immigration law and policy will be held strictly separate from health law and access to health care, i.e. that immigration status will not affect access to health care.^7^ The authors note that the firewall argument has gained significant traction in discussions of migrant health because of the overwhelming evidence that migrants, for a variety of reasons (e.g. fear of police, fear of violence, racism, etc.), will often not seek medical attention because they believe that it will affect their residency status and that it may eventually lead to their deportation. This is particularly the case in instances when migrants are in a country illegally. And apart from sound compassionate reasons, it is instrumentally important to address infectious diseases like TB promptly to protect one’s own citizens. As such, it is vital that governments make efforts to separate immigration law from healthcare, and it is important that they convey this message as best as possible within migrant communities through community outreach and community engagement practices.

## Migrants and TB in Canada

In 2017, 1796 people were diagnosed with active TB in Canada; with an incidence rate of 4.8 cases per 100 000 population, it is considered a low-incidence setting. Migrants are disproportionately represented among people with TB, where seven in every ten people with TB (*n* = 1123) are born outside Canada. Nationally, migrants diagnosed with TB come from all nine of the WHO epidemiological regions; however, persons born in five countries account for 60% of TB diagnoses in migrants to Canada: India, Philippines, China, Vietnam and Pakistan.^17^ Most active TB in migrant populations results from ‘reactivation’ of LTBI acquired prior to immigration to Canada. LTBI reactivation can occur any time post-immigration, with the highest TB incidence in the first few years post-arrival.^18^

At present, prospective permanent residents to Canada and select students, visitors, and temporary workers (i.e., those who stay in Canada for >6 months) undergo the immigration medical exam, which includes screening for active TB prior to immigration.^19^ People diagnosed with active TB during pre-immigration screening must complete a full course of active TB therapy prior to moving to Canada. Post-immigration surveillance is performed on select individuals considered at high risk for active TB, such as people with prior active TB or chest X-rays findings compatible with healed TB. These individuals are referred for follow-up with provincial health authorities, and although adherence with post-immigration surveillance is not mandatory for maintaining permanent residency status, it is our experience that it may be mistakenly perceived as a mandatory step by migrants. Otherwise, there are no national pre- or post-immigration LTBI screening and treatment programs. Moreover, migrants are covered under each provinces’ healthcare plans once they have established residence for at least 3 months; prior to that, however, it remains unclear what general medical coverage they do receive as there are some reports that non-governmental organizations are trying to fill that void in the interim,^20^ although TB care would still be provided regardless.

## Ethical challenges for migrants with TB in Canada

Two of the main ethical issues faced by those working in TB care and public health in Canada center on (i) inter- and intra-jurisdictional issues (i.e., between Canada and other countries, and within Canada, respectively), and (ii) navigating power imbalances between healthcare workers and migrants with TB. We will describe each issue in turn.

### Jurisdictional challenges

Across the provinces of Canada, we tend to see the following categories of migrants: economic migrants, family class migrants and temporary migrants (i.e. visitors/students/workers). Economic migrants generally have stable or substantive financial resources and have relocated for self-perceived better business or economic opportunities. Migrants in this category tend to be from TB endemic countries and may potentially travel frequently between Canada and their region of emigration. This raises a host of interjurisdictional issues between the rights and obligations of migrants and healthcare workers in Canada. Namely, what obligation do physicians, public health professionals, and public health agencies in Canada have to relay information about migrants’ TB status back to their countries of origin? On the one hand, the privacy and confidentiality of such migrants with TB must be upheld if they are non-infectious and have access to treatment, such that one might argue against disclosing a person’s TB status. Moreover, it is not clear how such information will be used by a foreign country and what constraints may be placed upon a returning individual. For example, provincial public health authorities in Canada will often not allow a client with TB to fly back to Canada if they know the client is not on the required medication.^21^ On the other hand, as members of a global community struggling to curtail and eliminate TB, the duty to protect others from the harm of TB infection and potential disease is also paramount. Currently, if the person with TB is adherent to treatment, we do not routinely alert overseas colleagues in order to uphold their privacy; however, we advise persons with TB that it is best to complete their treatment prior to traveling. However, if we believe adherence may be an issue, we tend to err on the side of providing countries information when we know that a person with TB will return home; we trust that our colleagues in these other countries will do their best to protect individuals from stigma and discrimination often associated with TB and that they will provide the best level of care that is available locally.

Jurisdictional challenges also exist within Canadian borders, both between provinces and between the provinces and the federal government. Constitutionally, decision-making authority regarding public health and healthcare is the purview of the provinces in Canada (with some exceptions not directly relevant for this paper), while the federal government retains power regarding the matter of immigration. As such, federal policy regarding TB screening for migrants lies with the federal government, while the provinces are responsible for caring for those migrants living with LTBI or active TB disease. Challenges may still exist when there is discordance between federal policy and provincial capacity, e.g. if federal immigration policy changes place a greater services burden onto provinces without requisite funding. It is the experience of the authors that the provinces and the federal government generally work and are in frequent dialog in a spirit of solidarity for the health of migrants and the protection of the general Canadian public. As such, the provinces and the federal government in Canada work together to ensure that federal laws and policies do not prejudice migrants with health issues, including TB, and vice versa.

### Power imbalances and the firewall argument

Another category of migrants common to BC and Canada are ‘temporary foreign workers’, who are often migrants who come to Canada in order to work and earn money in order to return to their country of origin with greater financial resources or to send money back to family abroad. There is often a power imbalance, real or perceived, between temporary foreign workers and their employers, as the workers are dependent upon their employers for work and remaining in in Canada. They may also be afraid of government authorities, worrying about their immigration status. Temporary foreign workers who come from TB endemic countries and who progress from LTBI to active disease while in Canada may be hesitant to seek care for fear they will be deported and lose their jobs; anecdotally, we know this has happened in the past though such cases are never officially recorded, e.g. nannies being fired for having TB by local families who are their employers. Although their seeking healthcare in general, and TB care in particular, in no way affects their immigration status, the perception that it does raises clinical and public health challenges, including potentially losing them to follow-up care. To return to the firewall argument from above, it becomes imperative to communicate with migrants, like temporary foreign workers, that their status in Canada will not be adversely affected by seeking care. The longstanding tradition in Canada—based in part (but not exclusively) on the separation of powers between the federal government, who is in charge of immigration, and the provincial governments, who are in charge of health—is to care for migrants in instances of clinical emergencies and when there are potential ill-effects on the public’s health. As such, healthcare workers, but more importantly, provincial governments and the Canadian government, must take steps to ensure that migrants understand that seeking medical care is appropriate and safe. For example, public health units might work with local organizations that help support new migrants in Canada, or the federal government can strive to clearly express the division between immigration law and public health in materials provided to persons in their countries of origins and when they arrive. Moreover, public health programs across Canada must have the budget sufficient to provide social protections (e.g. food, safe shelter, etc.), including protecting the clients from potential catastrophic costs, something that is currently not in place in many Canadian jurisdictions but which is part of the WHO’s ‘End TB Strategy’*.*^16^

## Conclusion

The challenges faced by migrants who immigrate to Canada—and perhaps similar HIC with universal healthcare—while suffering from TB are, luckily, more tractable than those faced by refugees who are escaping war and other forms of violence. However, the ethical challenges that arise in the care of economic migrants, family class migrants and temporary foreign workers require attention too. Given the federated, decentralized health care system we have in Canada, greater attention must be given to ensure that those who are new to Canada and suffer from LTBI or active TB are given the proper care, including access to social protections, while being explicitly reassured that seeking access to healthcare will not impact on their immigration status, and deploying the necessary recourses in situations when a person’s immigration status is inadvertently and inappropriately affected. How the issues faced by TB workers with regards to migrants in Canada relates to challenges faced by other TB workers and migrants in other HIC is yet to be determined. In part, this is an empirical question that suggests opportunities for future social science research (e.g. case study comparisons) between various HIC immigration and TB policies. At the very least, the issues identified herein serve to continue the dialog around the ethical treatment of migrants with TB.

## Notes

**Diego S. Silva**, Lecturer

**Victoria J. Cook**, Medical Lead

**James C. Johnston**, Evaluation Lead

**Jennifer Gardy,** Deputy Director
